# An Unusual Presentation of Nocardiosis in an Allogeneic Transplant Recipient

**DOI:** 10.7759/cureus.834

**Published:** 2016-10-17

**Authors:** Uroosa Ibrahim, Amina Saqib, Farhan Mohammad, Terenig Terjanian

**Affiliations:** 1 Department of Hematology/Oncology, Staten Island University Hospital; 2 Pulmonary/Critical Care, Staten Island University Hospital; 3 Medicine, Staten Island University Hospital

**Keywords:** nocardiosis, stem cell transplantation, brain abscess

## Abstract

Nocardiosis is a rare cause of opportunistic infection post hematopoietic stem cell transplant (HSCT) occurring in about 0.3% of patients. The risk factors include delayed immune reconstitution, prolonged neutropenia, and graft-versus-host disease. The most common site of infection is the lung, followed by the brain and the skin. Concomitant pulmonary and central nervous system (CNS) nocardiosis is an extremely rare entity as presented in our case. We present the case of a 72-year-old male at 137 days post transplant presenting with complaints of headache and slurred speech. A magnetic resonance imaging (MRI) brain scan revealed two ring-enhancing lesions: 1.6 cm in the right frontal lobe and 1 cm in the left parietal lobe. The patient had an outpatient computed tomography (CT) chest scan a month prior showing a 1.4 cm solid right upper lobe nodule prompting bronchoalveolar lavage (BAL) that was nondiagnostic. On repeat inpatient CT chest scan, the nodule had increased in size to 3.3 x 2.5 x 2.1 cm, prompting a percutaneous fine-needle aspiration biopsy. He was started on empiric trimethoprim-sulfamethoxazole (TMP-SMX) and liposomal amphotericin B. The tissue mycology and acid-fast cultures were reported positive for nocardia species. The patient was discharged on intravenous TMP-SMX. A follow-up CT chest scan and MRI brain scan four months later showed resolution of the right upper lobe nodule and significant decrease in size of the brain lesions. The patient will continue TMP-SMX for a total of nine to 12 months. Given the increase in transplant recipients and the ongoing risk of developing nocardiosis several months post transplant, there is a need for standardized diagnostic and treatment guidelines. Meanwhile, our case highlights the importance of aggressiveness in pursuing a prompt diagnosis including invasive procedures, if required, in order to begin specific treatment.

## Introduction

Nocardia species are an extremely rare cause of opportunistic infection in the setting of delayed immune reconstitution following hematopoietic stem cell transplant. Pulmonary nocardiosis is the most common site, and concomitant lung and CNS infection is an extremely uncommon entity. Predisposed individuals include those not on prophylaxis while in the immunocompromised state, such as transplant recipients, patients on immunosuppressive therapy and human immunodeficiency virus-infected individuals. A high index of clinical suspicion must exist in order to promptly diagnose the condition and initiate treatment, which may need to be long term. We report a case of pulmonary and CNS nocardiosis diagnosed on tissue biopsy culture with evidence of radiologic treatment response on intravenous trimethoprim-sulfamethoxazole. Informed consent was obtained from the patient for the treatment.

## Case presentation

A 72-year-old male presented to our hospital with symptoms of an acute headache and slurred speech. His past medical history included hypertension, hypothyroidism, and a recent diagnosis of left superficial femoral and popliteal vein thrombosis. He was known to our hematology/oncology service for a diagnosis of blastic plasmacytoid dendritic cell neoplasm treated with chemotherapy and allogeneic stem cell transplant (day 137 at presentation). He was on rivaroxaban for the deep vein thrombosis (DVT), tacrolimus for graft-versus-host disease (GVHD) prophylaxis as well as standard post-transplant antimicrobial prophylaxis. On examination, he had decreased strength in his bilateral lower extremities with a power of 4/5. The rest of the examination was unremarkable. He was an ex-smoker with social alcohol use. The laboratory findings were consistent with pancytopenia at his baseline and a therapeutic tacrolimus level.

**Figure 1:**
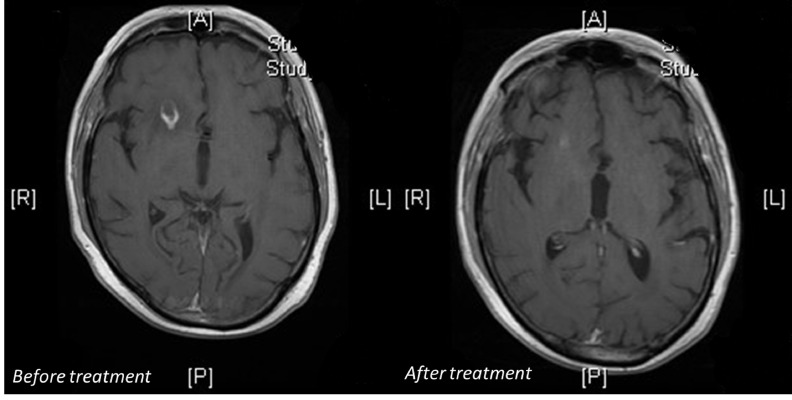
Right frontal lobe ring-enhancing 1.6 cm lesion before and after treatment

**Figure 2:**
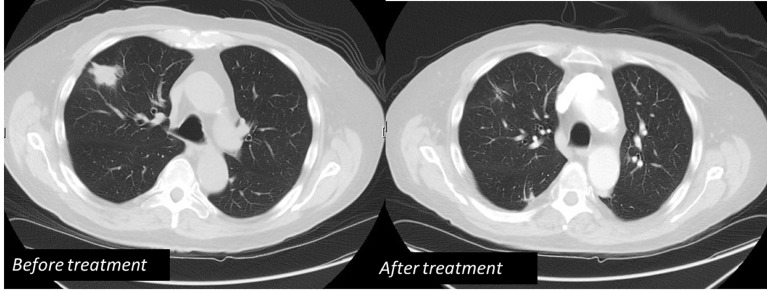
Right upper lobe 3.3 cm solid nodule before and after treatment

The patient’s neurologic symptoms prompted an MRI of the brain that revealed two ring-enhancing lesions: a 1.6 cm lesion in the right frontal lobe and a 1 cm lesion in the left parietal lobe, both with surrounding vasogenic edema (Figure 1). A review of the patient’s records revealed that he had a CT chest scan a month prior to presentation for follow-up of a pulmonary nodule that showed a new 1.4 cm solid right upper lobe nodule. This was followed by bronchoscopy and bronchoalveolar fluid examination that was negative for gram stain, fungal, pneumocystis and acid-fast stains and cultures. Clusters of acute inflammatory cells were seen; no malignant cells were present. The patient’s inpatient CT chest scan revealed enlargement of the previous nodule, now measuring 3.3 x 2.5 x 2.1 cm, prompting a CT-guided fine-needle aspiration biopsy (Figure 2). The pathology was consistent with inflammatory changes including abscess formation and focal necrosis. The specimen was sent for bacterial, fungal, and mycobacterial cultures and cytology. Meanwhile, the patient also had a lumbar puncture with cerebrospinal fluid (CSF) analysis that failed to reveal any abnormality. The CSF culture did not show any growth. Serology for toxoplasma, histoplasma, coccidioides and cryptococcus was negative. The patient was started on empiric therapy with intravenous TMP-SMX and liposomal amphotericin B along with dexamethasone for vasogenic brain edema. The tissue mycology culture was reported positive for nocardia species. The acid-fast stain was negative, and the acid-fast culture was also positive for nocardia species. The patient was discharged on intravenous TMP-SMX with a peripherally inserted central catheter (PICC) in place.

A follow-up CT chest scan and MRI brain scan four months later showed resolution of the right upper lobe nodule with residual scarring and decrease in size of the ring-enhancing brain lesions: right frontal lobe lesion 7 mm down from 1.6 cm and left parietal lobe lesion 8 mm down from 1 cm. The patient will continue TMP-SMX for a total of nine to 12 months.

## Discussion

Despite advances in antimicrobial therapy, infections remain the major cause of life-threatening complications in patients with hematopoietic cell transplantation. Patients at highest risk of nocardia infection are those with prolonged neutropenia, those on immunosuppressive therapy, those with acute or chronic graft-versus-host disease and lack of TMP-SMX prophylaxis. Other risk factors include protracted steroid use, antilymphocyte antibody treatment, and a high calcineurin inhibitor level in the preceding 30 days [1].

Nocardia species are fastidious aerobic gram-positive organisms that are typically partially acid-fast. Over 80 species have been described with approximately half of those responsible for human disease [2]. Although exceedingly rare, with an incidence of 0.3% to 1% [3], nocardia infections have been observed in allogeneic transplant recipients in multiple sites including the lungs, brain, and skin [4]. A decline in the incidence has been seen possibly because of TMP-SMX prophylaxis and earlier identification and management of transplant-associated complications. Among HSCT recipients, most cases have been described in allogeneic recipients although it may occur after an autologous transplant. The time lapse from HSCT to infection has been noted to be between six to 10 months. However, nocardiosis has been diagnosed up to 15 years after allogeneic HSCT [5].

Nocardiosis may present as localized or disseminated disease. When at least two sites are involved, the disease is considered disseminated. Pulmonary nocardiosis is the most common form of nocardia infection occurring in about 39% of cases. It may manifest on imaging studies as a consolidation, nodules, cavitary lesion, or pleural thickening [6]. Symptoms, if present, may include nonproductive cough and pleuritic chest pain. Central nervous system nocardiosis usually manifests as a parenchymal abscess appearing as a ring-enhancing lesion on a CT scan or MRI. The MRI helps differentiate an abscess from a neoplastic lesion [7]. The symptoms are nonspecific and may include fever, headache, seizures or focal neurologic deficits [8].

A definitive diagnosis of nocardiosis requires isolation of the pathogen from culture of the involved site. This often requires an invasive procedure and must be pursued when a high degree of suspicion exists. With suspected pulmonary nocardiosis, BAL is performed because of the high frequency of coinfections and broad differential diagnosis. When BAL is nondiagnostic, a biopsy must be considered [9]. In cases of suspected CNS infection, prompt radiological workup must be pursued because of the nonspecific symptoms and slow symptom evolution [10].

Three cases of concomitant CNS and pulmonary nocardiosis have been described in the English literature. One of them was diagnosed with a pulmonary biopsy and the other two were based on biological and radiological arguments [3]. All three of them were treated for at least 10 months. The treatment of choice for nocardia infection remains TMX-SMX. Other agents that can be used include imipenem, minocycline and amikacin. Given the rarity of the disease, treatment recommendations are based on retrospective studies and case reports. In immunosuppressed hematopoietic cell transplant recipients, treatment is generally continued for six to 12 months [10]. Close follow-up is desired to assess for response as well as to monitor for other opportunistic infections in these high-risk patients.

## Conclusions

Although rare, nocardiosis can be a life-threatening infection in susceptible individuals. Prospective studies are lacking to guide treatment decisions. Given the increasing number of solid organ and hematopoietic cell transplants being performed and the ongoing risk of developing the infection several months post transplant, there is a need for standardized diagnostic and treatment guidelines. Meanwhile, when infection is suspected, aggressive efforts must be made to reach a prompt diagnosis and initiate therapy.
